# Supplementary material for: Therapeutic effects of telomerase in mice with pulmonary fibrosis induced by damage to the lungs and short telomeres
Source: eLife. 2018 Jan 30;7:e31299. doi: 10.7554/eLife.31299 (PMC5818250; doi:10.7554/eLife.31299)
Supplement: Supplementary file 1. — The FDR is calculated by Benjamini and Hochberg FDR correction. [file elife-31299-supp1.docx]

**Supplementary Table 1.** Differentially expressed genes in AAV9-*Tert* compared to empty vector treated fibrotic lungs (FDR<0.05). The FDR is calculated by Benjamini and Hochberg FDR correction.

| **Gene symbol** | **GeneName** | **adj.P.Val** |
| --- | --- | --- |
| *Apol9a* | apolipoprotein L 9a | 0.0015 |
| *Oas2* | 2'-5' oligoadenylate synthetase 2 | 0.0015 |
| *Apol9a* | apolipoprotein L 9a | 0.0015 |
| *Apol9b* | apolipoprotein L 9b | 0.0015 |
| *Gm4955* | predicted gene 4955 | 0.0015 |
| *Irf7* | interferon regulatory factor 7 | 0.0015 |
| *Ifi27l2a* | interferon, alpha-inducible protein 27 like 2A | 0.0017 |
| *EG545385* | predicted gene, EG545385 | 0.0024 |
| *Oas1a* | 2'-5' oligoadenylate synthetase 1A | 0.0030 |
| *Isg15* | ISG15 ubiquitin-like modifier | 0.0032 |
| *Oas1a* | 2'-5' oligoadenylate synthetase 1A | 0.0032 |
| *Ifi44* | interferon-induced protein 44 | 0.0032 |
| *Ifit1* | interferon-induced protein with tetratricopeptide repeats 1 | 0.0045 |
| *Mx1* | myxovirus (influenza virus) resistance 1 | 0.0053 |
| *Zbp1* | Z-DNA binding protein 1 | 0.0056 |
| *Oas1f* | 2'-5' oligoadenylate synthetase 1F | 0.0057 |
| *Zbp1* | Z-DNA binding protein 1 | 0.0083 |
| *Isg15* | ISG15 ubiquitin-like modifier | 0.0083 |
| *Mx2* | myxovirus (influenza virus) resistance 2 | 0.0083 |
| *LOC100048309* | similar to interferon activated gene 204 | 0.0083 |
| *Oasl1* | 2'-5' oligoadenylate synthetase-like 1 | 0.0093 |
| *Phf11* | PHD finger protein 11 | 0.0099 |
| *Oasl2* | 2'-5' oligoadenylate synthetase-like 2 | 0.0102 |
| *Ccl4* | chemokine (C-C motif) ligand 4 | 0.0117 |
| *Plac8* | placenta-specific 8 | 0.0117 |
| *Oas3* | 2'-5' oligoadenylate synthetase 3 | 0.0123 |
| *Ifi204* | interferon activated gene 204 | 0.0132 |
| *Nkg7* | natural killer cell group 7 sequence | 0.0183 |
| *Plac8* | placenta-specific 8 | 0.0191 |
| *Csprs* | component of Sp100-rs | 0.0217 |
| *Klra23* | killer cell lectin-like receptor subfamily A, member 23 | 0.0219 |
| *Slfn2* | schlafen 2 | 0.0303 |
| *Klra23* | killer cell lectin-like receptor subfamily A, member 23 | 0.0312 |
| *Lgals3bp* | lectin, galactoside-binding, soluble, 3 binding protein | 0.0315 |
| *Ifng* | interferon gamma | 0.0354 |
| *Klra16* | killer cell lectin-like receptor, subfamily A, member 16 | 0.0364 |
| *Klra7* | killer cell lectin-like receptor, subfamily A, member 7 | 0.0364 |
| *Mefv* | Mediterranean fever | 0.0364 |
| *LOC635676* | hypothetical protein LOC635676 | 0.0364 |
| *Slfn4* | schlafen 4 | 0.0364 |
| *Cmpk2* | cytidine monophosphate (UMP-CMP) kinase 2, mitochondrial | 0.0371 |
| *LOC100041903* | similar to putative G-protein coupled receptor | 0.0375 |
| *Klra22* | killer cell lectin-like receptor subfamily A, member 22 | 0.0396 |
| *C79246* | expressed sequence C79246 | 0.0396 |
| *Ccl5* | chemokine (C-C motif) ligand 5 | 0.0396 |
| *LOC631406* | similar to schlafen 8 | 0.0414 |
| *Fasl* | Fas ligand (TNF superfamily, member 6) | 0.0435 |
| *Pyhin1* | pyrin and HIN domain family, member 1 | 0.0438 |
| *Ms4a6c* | membrane-spanning 4-domains, subfamily A, member 6C | 0.0456 |
| *Ms4a4c* | membrane-spanning 4-domains, subfamily A, member 4C | 0.0482 |
| *D14Ertd668e* | DNA segment, Chr 14, ERATO Doi 668, expressed | 0.0482 |
| *Klra23* | killer cell lectin-like receptor subfamily A, member 23 | 0.0493 |
| *Gm4902* | predicted gene 4902 | 0.0493 |
